# Sexual diversity in the United States: Results from a nationally representative probability sample of adult women and men

**DOI:** 10.1371/journal.pone.0181198

**Published:** 2017-07-20

**Authors:** Debby Herbenick, Jessamyn Bowling, Tsung-Chieh (Jane) Fu, Brian Dodge, Lucia Guerra-Reyes, Stephanie Sanders

**Affiliations:** 1 Center for Sexual Health Promotion, School of Public Health, Indiana University, Bloomington, Indiana, United States of America; 2 College of Health & Human Services, University of North Carolina, Charlotte, North Carolina, United States of America; 3 Department of Gender Studies, Indiana University, Bloomington, Indiana, United States of America; China Medical University, CHINA

## Abstract

In 2015, we conducted a cross-sectional, Internet-based, U.S. nationally representative probability survey of 2,021 adults (975 men, 1,046 women) focused on a broad range of sexual behaviors. Individuals invited to participate were from the GfK KnowledgePanel^®^. The survey was titled the 2015 Sexual Exploration in America Study and survey completion took about 12 to 15 minutes. The survey was confidential and the researchers never had access to respondents’ identifiers. Respondents reported on demographic items, lifetime and recent sexual behaviors, and the appeal of 50+ sexual behaviors. Most (>80%) reported lifetime masturbation, vaginal sex, and oral sex. Lifetime anal sex was reported by 43% of men (insertive) and 37% of women (receptive). Common lifetime sexual behaviors included wearing sexy lingerie/underwear (75% women, 26% men), sending/receiving digital nude/semi-nude photos (54% women, 65% men), reading erotic stories (57% of participants), public sex (≥43%), role-playing (≥22%), tying/being tied up (≥20%), spanking (≥30%), and watching sexually explicit videos/DVDs (60% women, 82% men). Having engaged in threesomes (10% women, 18% men) and playful whipping (≥13%) were less common. Lifetime group sex, sex parties, taking a sexuality class/workshop, and going to BDSM parties were uncommon (each <8%). More Americans identified behaviors as “appealing” than had engaged in them. Romantic/affectionate behaviors were among those most commonly identified as appealing for both men and women. The appeal of particular behaviors was associated with greater odds that the individual had ever engaged in the behavior. This study contributes to our understanding of more diverse adult sexual behaviors than has previously been captured in U.S. nationally representative probability surveys. Implications for sexuality educators, clinicians, and individuals in the general population are discussed.

## Introduction

Alfred Kinsey and colleagues documented sexual diversity in the United States (U.S.) in large convenience samples of thousands of women and men in the 1930s – 1950s [1, 2]. The great interest with which their team’s findings were met by the American population and scientific community emphasize the value of studying sexual behavior and its many expressions. Because sexual behaviors are often private, and because sexuality topics are often shrouded in secrecy and taboo, sexuality remains a particularly important topic to highlight in scientific research in order to expand knowledge and understanding of this important aspect of human life.

Although Kinsey’s team asked about a broad range of sexual behaviors, few adults older than age 50 were interviewed and U.S. probability sampling was not then feasible. Thus, although human sexual behaviors were established as diverse, the population-based *prevalence* of such behaviors was unknown. Consequently, while sexual health professionals have long been able to utilize Kinsey’s data to reassure or inform clients, patients, policy makers, and one another that, indeed, humans express their sexual desires and interests through diverse sexual behaviors, just how rare or how common many sexual behaviors are has been unknown. All too often, when asked how common certain sexual expressions are, sexuality professionals have had to respond, “We don’t know.”

The National Health and Social Life Survey (NHSLS), conducted in 1991, was the first nationally representative survey of U.S. sexual behavior, providing population estimates of a limited range of sexual behaviors. The NHSLS answered some of these important questions (particularly those related to the prevalence of masturbation, vaginal intercourse, anal intercourse, oral sex, and the appeal of a range of sexual experiences), yet the survey was also limited to younger adults (ages 18–59) [3].

In addition to the NSHLS, there have been several important U.S. national studies that have addressed sexual behavior, though each has focused on a narrow range of ages and/or sexual behaviors. The National Survey of Family Growth (NSFG) and Youth Risk Behavior Survey (YRBS) survey younger age groups and have mostly asked about sexual behaviors related to risk of pregnancy and sexually transmitted infections (STI) [3, 4]. Also, YRBS items pertaining to sexual behavior are not uniformly adopted by all U.S. states [4]. The National Social Life, Health, and Aging Project (NSHAP) addresses a limited scope of relational and sexual issues of older Americans [5].

The National Surveys of Sexual Health and Behavior (NSSHB) are the most recent U.S. probability surveys focused specifically on human sexual expression and have sampled a broad spectrum of Americans including adolescents as young as age 14 and adults in their 80s and 90s [6–14]. Although the NSSHB assesses a fairly broad range of sexual behaviors among a range of ages, the focus has still largely been on solo and partnered masturbation, oral sex, vaginal sex, anal sex, digital penetration of the vagina and anus, and the use of condoms, lubricants, and sex toys. NSSHB items are broader in behavioral scope than the United Kingdom’s National Surveys of Sexual Attitudes and Lifestyles (NASTAL) [15] and somewhat more limited in behavioral scope than the Australian Survey of Health and Relationships which has—among Australians ages 16 to 69—also asked respondents about fisting, rimming, role play or dressing up, and group sex [16, 17].

U.S. data on diverse sexual behaviors are needed to improve clinicians’, educators’, policymakers’ and the general public’s understanding of human sexual expression. Consequently, human sexual expression can be more richly and accurately described which may also help people to feel “seen” or better represented in terms of their sexualities. Occasional population-based benchmarks of sexual behaviors are also helpful in understanding whether certain cultural moments influence sexual behavior. For example, the popularity of the book *Fifty Shades of Grey* prompted journalists to ask how many people may feel aroused by, or participate in, bondage, domination, submission/sadism, and masochism (BDSM)—also referred to as “kink” behaviors [18, 19]—and whether reading the book changed population-level sexual behavior. In fact, the book series and 2015 film release have been linked to increases in rope sales in hardware stores [20], sex toy sales [21, 22], as well as BDSM sex toy-related injuries [23]. However, since there had been no earlier national benchmarks of BDSM-related behaviors in the U.S., it was impossible to assess whether sexual practices had indeed changed at the population level.

That said, there have been a number of other societal level changes relevant to Americans’ sexual practices such as greater acceptance of same-sex partnerships and marriage and more representations in mainstream media of open relationships and marriages [24, 25]. Technological innovations have resulted in greater access to sexually explicit material and greater ease of taking and sharing sexually explicit photographs and videos. [We note that these innovations are available in many countries globally; however, because the present research is limited to Americans’ sexual behaviors, we focus our discussion of influencing factors mostly on those likely to be experienced by individuals living in the U.S. at the time of the study.] Although humans have engaged in a wide range of sexual behaviors throughout historical time and place, researchers have yet to document such diverse behaviors in a U.S. population-based sample. Using data from the 2015 *Sexual Exploration in America Study*, a nationally representative probability sample of U.S. adults ages 18 and over, we sought to estimate the prevalence of a broad and diverse range of sexual behaviors of American women and men as well as to understand more about the appeal of such behaviors.

## Materials & methods

### Data collection

The Institutional Review Board (IRB) of the Human Subjects Office at Indiana University-Bloomington reviewed and approved all study protocols (#141211166). Data were collected in late December 2014 and January 2015 through the KnowledgePanel^®^ of GfK Research (GfK) (Menlo Park, CA, USA). Research panels accessed through GfK are based on a U.S. nationally representative probability sample established using both random digit dialing and, since 2009, an address-based sampling (ABS) frame that covers about 97% of U.S. households including unlisted telephone numbers, those without landlines, those that are cell phone only, and those without Internet access. [26] Non-Internet households selected into the sample are provided hardware (i.e., a web-enabled computer) and Internet service to facilitate participation. The GfK KnowledgePanel^®^ has been utilized in numerous nationally representative probability surveys, including those related to sexual behaviors and health, as well as other topics (see for example: [27, 28–34]). GfK panel members are invited to take a small number of surveys per month (often just one or two) and can earn points for completing surveys that they can then accumulate and exchange for products. No additional incentives were offered for completing of the present survey.

Once the sample frame of adults 18+ was established, 4532 individuals were sent an email from GfK informing them that a new survey was available. Those who clicked on the link to learn more about the survey were provided with a brief description of the survey topic and the IRB-approved Study Information Sheet; a total of 2021 individuals (47% of those invited) consented to participate and completed the survey. The Study Information Sheet described the research study as being “about the romantic relationships and sexual behaviors of adults in the United States”, noted that people were being asked to participate even if they had never had sex or even if it had been a long time since they last had sex, described the confidentiality of the survey, and noted that participants could skip any questions they did not wish to answer. In order to normalize a range of responses, we also noted in the Study Information Sheet (as we often do in related research) that while some people find it “embarrassing or uncomfortable” to be asked about their sexual behaviors, others “find it interesting to think about their romantic and sexual experiences”.

### Measures

Panel members are asked by GfK to complete demographic items on an annual basis for sampling stratification and post-sampling weighting purposes; thus items related to age, gender, race/ethnicity, education, marital status, household income, and employment status were provided by GfK and not asked again by the research team. In addition, questions regarding perceived happiness and health status (from the General Social Survey) were asked.

#### Relationship structure

We asked respondents to indicate their relationship status; those who were in a relationship were asked whether that relationship was—during the past year—*entirely monogamous* (partners agreed to have sex only with each other and indeed only being sexual with each other to the respondent’s knowledge), *monogamous but sexless* (partners agreed to be “exclusive” with each other but did not have sex together in the past year), *supposedly monogamous* (had agreed to be sexual only with each other and one or both partners had engaged in sex with others), in an *open relationship* (had agreed that one or both partners would engage in sexual activities with others); or had *not discussed* their relationship structure. Another option was to describe it some other way, with a text box offered.

#### Sexual orientation

Consistent with the NSSHB item about sexual orientation [9], respondents were asked, “Which of the following best describes your sexual orientation?” (*heterosexual/straight*, *gay or lesbian*, *bisexual*, *asexual (not sexually attracted to others)*, *other/please describe*).

#### Sexual behaviors

Respondents were also asked how recently they had engaged in 32 sexual behaviors using a response scale common to the NSSHB [8] (*past month*, *past year*, *more than a year ago*, *never*) for the purposes of context and, where applicable, comparison. The sexual behavior items were developed by the first author with feedback from four American masters- and doctoral-level individuals who have engaged extensively with and/or identify with communities related to BDSM, swinging, sex parties, group sex, and other forms of sexual diversity and/or kink. Item order within this section was randomized. Prior to asking these items, participants read a screen that said:

“In this next section we will ask you questions about many different things that people do in their sexual lives. Some are common and many people do them. Others may be less common. Your responses will help us to better understand Americans’ sexual lives. Remember: your answers are completely confidential.”

In measuring the appeal of more than 50 sexual behaviors, the response options used in the NHSLS [35] were presented to participants (*very appealing*, *somewhat appealing*, *not appealing*, *not at all appealing*) to facilitate comparison for the few parallel items. Item order within this section was randomized.

### Statistical analyses

Analyses were conducted using SPSS version 22.0 (IBM Corp, 2013). A general population weight (calculated and provided by GfK) was applied to the data in order to minimize bias and variance due to nonsampling error. Weighting was calculated based on the March 2014 supplement of the Current Population Survey (CPS) with variables such as gender, race/ethnicity, age, education, and household income.

Given the large number of sexual behaviors asked about, for purposes of creating more manageable tables, we grouped the sexual behaviors as solo and partnered sexual behaviors (e.g., masturbation, vaginal sex, oral sex, wearing sexy lingerie/underwear for a partner), those involving the use of sexual enhancement products and/or media (e.g., using sex toys, sharing nude images via Text, watching sexually explicit media), and, for lack of a better term, “social” sexual behaviors (e.g., threesomes, group sex, sex parties). These groupings are utilized solely for presentation in the present manuscript and do not reflect the order items were asked in the survey, which was randomized as described earlier.

Chi-squared tests were used to identify gender differences for the 32 sexual behaviors and the appeal of 50+ sexual behaviors. Our goal was to set the overall familywise error rate at 0.05 or less. Using the Bonferroni correction, we thus needed to use a p-value of .0016 (0.05/32) and .0009 (0.05/53), respectively, for each comparison; an alpha of .001 was used for these analyses.

Sexual behaviors were dichotomized to “never” and “lifetime” of ever having engaged in it. Appeal variables were dichotomized to “not appealing” (including “not appealing” and “not at all appealing”) and “appealing” (including “somewhat appealing” and “very appealing”). Multivariate logistic regression was used to examine the effect of appeal on having engaged in the relevant behavior. For example, the appeal of oral sex was examined in relation to whether participants had actually engaged in the behavior. The dichotomized behavior variable was the outcome, and the corresponding dichotomized appeal variable was the predictor of interest. All models were adjusted for age (categorical; 18-24/25-29/30-39/40-49/50-59/60-69/70+), perceived health status (categorical; poor/fair/good/very good/excellent), relationship status (categorical; single/in a relationship/married) and relationship duration (continuous; years). Results generating p-values less than 0.05 were considered statistically significant.

## Results

The sample included 975 men and 1046 women (see Table 1 for demographic information and presentations of both unweighted and weighted total sample) with a mean age of 47.1 (SD = 17.3; range = 18–91). About 91% identified as heterosexual, with more women identifying as bisexual (3.6%) compared to lesbian (1.5%) and more men identifying as gay (5.8%) compared to bisexual (1.9%). Most respondents reported being generally “very happy” or “pretty happy” (88%), and nearly 86% reported “good”, “very good”, or “excellent” general health. Of those in relationships, most were in male-female romantic relationships (95.2% men, 96.8% women). About half were married.

**Table 1:** Weighted demographic characteristics of participants by gender.

	Unweighted Total (N = 2021)	Weighted Total (N = 2021)	Men (N = 975)	Women (N = 1046)
	*% (n)*	*% (n)*	*% (n)*	*% (n)*
**Age**				
18–24	9.2 (186)	11.0 (221)	12.5 (122)	9.5 (99)
25–29	9.4 (189)	10.5 (211)	9.8 (95)	11.1 (116)
30–39	14.1 (285)	15.6 (315)	15.1 (147)	16.0 (167)
40–49	16.1 (326)	16.6 (336)	17.5 (171)	15.7 (164)
50–59	22.5 (455)	20.0 (404)	20.1 (196)	19.8 (208)
60–69	16.3 (330)	15.0 (304)	13.1 (128)	16.8 (176)
v≥70	12.4 (250)	11.4 (230)	11.7 (114)	11.1 (116)
**Race/Ethnicity**				
White, Non-Hispanic	71.2 (1438)	65.5 (1324)	65.9 (642)	65.1 (681)
Black, Non-Hispanic	9.9 (199)	11.5 (233)	10.8 (105)	12.3 (128)
Other, Non-Hispanic	3.5 (71)	6.5 (131)	6.3 (61)	6.7 (70)
Hispanic	12.5 (253)	15.2 (307)	15.8 (154)	14.7 (153)
2+ Races, Non-Hispanic	3.0 (60)	1.3 (26)	1.3 (12)	1.3 (13)
**Education**				
Less than high school	9.9 (200)	12.4 (250)	13.3 (129)	11.6 (121)
High school	29.9 (605)	29.6 (599)	30.7 (299)	28.7 (300)
Some college	27.5 (556)	28.7 (581)	27.3 (266)	30.1 (315)
Bachelor’s Degree or Higher	32.7 (660)	29.2 (591)	28.8 (281)	29.7 (310)
**Sexual Orientation**				
Heterosexual/straight	91.4 (1847)	91.0 (1840)	89.8 (875)	92.2 (965)
Bisexual (attracted to more than one gender)	2.8 (56)	2.8 (56)	1.9 (19)	3.6 (37)
Gay or lesbian	3.6 (72)	3.6 (72)	5.8 (57)	1.5 (14)
Asexual (not attracted to anyone)	0.8 (16)	0.9 (19)	0.5 (5)	1.3 (14)
Other	0.8 (17)	0.8 (16)	0.7 (7)	0.9 (10)
**Marital Status**				
Married	54.2 (1096)	51.0 (1030)	54.1 (528)	48.0 (503)
Widowed	4.4 (89)	4.3 (88)	1.7 (17)	6.8 (71)
Divorced	10.5 (212)	10.4 (211)	7.3 (71)	13.4 (140)
Separated	2.2 (44)	2.3 (46)	2.0 (19)	2.6 (27)
Never married	20.7 (419)	23.5 (475)	27.2 (265)	20.0 (210)
Living with partner	8.0 (161)	8.4 (171)	7.7 (75)	9.2 (96)
**Annual Household Income (USD)**				
<$25,000	17.4 (352)	19.0 (383)	17.2 (168)	20.6 (216)
$25,000–49,999	21.3 (431)	21.8 (441)	20.6 (201)	22.9 (240)
$50,000–74,999	18.7 (377)	18.4 (374)	19.0 (185)	18.0 (188)
≥$75,000	42.6 (861)	40.8 (824)	43.2 (421)	38.5 (403)
**Employment Status**				
Working—as a paid employee	52.5 (1060)	53.0 (1070)	55.5 (541)	50.6 (529)
Working—self-employed	5.6 (113)	5.5 (112)	6.5 (64)	4.6 (48)
Not working—on temporary layoff from a job	0.5 (11)	0.6 (11)	0.6 (6)	0.5 (5)
Not working—looking for work	6.8 (138)	7.9 (159)	9.3 (91)	6.5 (68)
Not working—retired	19.1 (386)	17.1 (346)	15.7 (153)	18.4 (193)
Not working—disabled	8.1 (163)	8.1 (165)	8.3 (81)	8.0 (84)
Not working—other	7.4 (150)	7.8 (158)	4.0 (39)	11.3 (119)

In thinking about the past year, most of the 1421 respondents who were in relationships reported being *entirely monogamous* (77.8%, n = 1106). More than 1 in 10 partnered respondents were currently in *monogamous but sexless* relationships (11.7%, n = 166). Additionally, 4.1% (n = 58) were *supposedly monogamous*, 1.6% (n = 23) reported being in an *open relationship*, 2.5% (n = 36) had *not discussed* their relationship structure, and the remaining 1.4% (n = 20) identified their relationship in some other way.

### Solo and partnered sexual behaviors

About 64% of men and 40.8% of women had masturbated in the last month and 8.2% of men and 21.8% of women reported having *never* masturbated in their lifetime. Vaginal intercourse and giving/receiving oral sex were the most common partnered behaviors (lifetime and recently; see Table 2) followed by partnered masturbation. The next most prevalent partnered lifetime behaviors differed by gender; for men it was having sex with someone in a public place (>45%) and for women it was wearing sexy underwear or lingerie for a partner (>75%). Significantly more women reported having engaged in lifetime vaginal sex (but not recent vaginal sex), receptive anal sex, and having worn sexy underwear for a partner. Significantly more men than women (25.6% vs. 10.9%) reported having ever engaged in sucking/licking of toes or feet. There were no statistically significant differences by gender in terms of spanking, whipping, partnered masturbation, role playing, tying up a partner or being tied up, giving or receiving oral sex, or having sex in public.

**Table 2:** Partnered sexual behaviors by gender and age (N = 2,021, Men: n = 975, Women: n = 1046).

			Men (%)								Women (%)						
	Total (%)	Total Men	18–24	25–29	30–39	40–49	50–59	60–69	70+	Total Women	18–24	25–29	30–39	40–49	50–59	60–69	70+
Vaginal intercourse*																	
Past month	52.4	**52.1**	39.3	68.9	63.2	62.3	49.1	44.1	37.2	**52.6**	52.1	72.6	77.3	62.0	52.8	32.6	14.6
Past year	64.1	**65.8**	53.3	74.4	74.9	79.3	65.6	57.5	50.6	**62.4**	62.1	83.6	85.3	74.2	64.5	39.3	23.8
Lifetime	88.5	**85.6**	59.4	87.4	85.0	90.2	90.6	93.2	87.4	**91.1**	65.8	90.9	92.7	92.5	95.0	94.1	96.1
Gave partner oral sex																	
Past month	34.9	**34.0**	32.9	50.7	41.5	34.0	36.8	21.7	21.4	**35.7**	44.7	53.5	53.0	41.6	31.9	19.7	8.0
Past year	54.6	**56.7**	49.5	69.4	71.3	67.9	57.7	42.3	33.7	**52.6**	57.3	76.4	73.8	63.0	54.3	27.4	15.2
Lifetime	82.7	**83.0**	61.3	84.8	88.2	88.4	87.9	86.8	76.1	**82.5**	62.3	85.5	89.0	89.0	86.4	85.1	66.9
Received oral sex																	
Past month	35.0	**38.4**	36.4	66.0	44.9	42.4	36.7	27.9	19.2	**31.8**	35.8	48.1	51.3	34.7	28.3	17.0	8.5
Past year	54.8	**60.9**	61.7	76.1	71.0	75.1	59.6	46.2	33.4	**49.2**	59.7	69.5	68.7	54.9	49.4	27.7	16.2
Lifetime	84.8	**85.0**	67.1	89.2	88.3	92.2	89.1	88.4	74.1	**84.6**	64.2	87.4	87.9	89.9	87.3	88.0	76.9
Insertive anal sex																	
Past month	-	**5.7**	2.7	8.1	10.1	8.7	6.3	1.4	0.8	-	-	-	-	-	-	-	-
Past year	-	**15.2**	12.4	21.1	26.1	21.9	12.7	7.5	3.4	-	-	-	-	-	-	-	-
Lifetime	-	**42.6**	20.2	44.3	55.1	57.9	45.3	35.5	29.7	-	-	-	-	-	-	-	-
Received anal sex*																	
Past month	3.5	**2.5**	7.2	3.3	3.9	0.4	2.2	0.7	1.4	**4.4**	6.7	9.9	5.0	5.0	5.0	0.6	0.0
Past year	8.7	**5.3**	8.9	5.6	10.5	7.0	2.6	1.2	1.4	**11.8**	17.0	25.5	18.3	14.7	9.8	1.2	0.0
Lifetime	23.8	**9.3**	9.8	5.6	15.4	11.3	7.7	7.2	6.4	**37.3**	27.7	41.2	46.2	44.2	39.9	33.6	20.1
Worn sexy underwear or lingerie for a partner*																	
Past month	10.3	**3.0**	3.9	8.4	2.3	1.4	3.8	1.8	0.8	**16.9**	23.9	29.5	27.0	15.1	16.3	7.2	2.6
Past year	22.7	**8.9**	8.2	17.7	10.1	10.4	8.6	6.4	1.9	**35.5**	45.9	63.3	55.0	36.1	31.7	15.1	7.1
Lifetime	51.8	**26.2**	13.5	23.5	30.8	33.3	33.9	28.1	10.1	**75.4**	61.9	81.0	85.2	75.0	80.8	72.8	61.4
Had sex with someone in public place																	
Past month	1.2	**1.5**	1.2	3.7	0.0	3.0	1.4	0.7	0.7	**0.9**	0.0	4.9	0.7	0.5	0.6	0.0	0.0
Past year	5.3	**6.0**	11.0	12.5	2.9	7.5	4.7	3.7	1.9	**4.7**	7.4	12.7	6.3	4.7	4.2	0.0	0.0
Lifetime	44.1	**45.4**	21.2	49.6	42.2	59.9	53.9	47.1	32.8	**42.9**	27.0	49.2	50.9	52.8	51.3	38.1	16.1
Tied up your partner or been tied up as part of sex																	
Past month	1.3	**1.4**	3.1	4.1	1.6	0.4	1.1	0.6	0.0	**1.3**	2.4	4.2	3.8	0.0	0.0	0.0	0.0
Past year	4.5	**4.3**	7.9	15.1	3.8	3.2	2.5	1.4	0.3	**4.8**	7.6	15.0	8.4	1.9	3.8	0.0	0.0
Lifetime	21.1	**21.7**	11.7	40.7	25.9	31.4	19.5	15.0	8.2	**20.6**	14.7	30.8	34.2	20.3	26.4	7.9	5.2
Sucked/licked partner’s feet/toes*																	
Past month	1.6	**2.3**	1.3	5.3	3.7	1.5	3.6	0.0	0.8	**0.9**	2.0	2.1	1.5	0.5	0.4	0.5	0.0
Past year	5.3	**8.3**	6.2	14.3	8.3	10.3	11.9	1.8	3.9	**2.6**	2.0	8.1	4.0	1.6	1.8	1.5	0.0
Lifetime	18.0	**25.6**	14.6	28.6	25.5	28.7	33.1	27.1	15.6	**10.9**	5.1	12.3	14.4	11.7	13.1	8.8	6.8
Masturbated with someone else (spouse, boy/girlfriend, friend, someone else)																	
Past month	18.8	**18.4**	21.9	19.6	18.8	24.0	19.0	13.7	9.6	**19.1**	21.6	30.9	30.8	24.3	16.5	9.3	0.7
Past year	33.2	**35.5**	35.8	43.0	49.4	48.3	28.9	25.1	16.8	**31.1**	27.6	50.7	51.7	38.1	29.9	13.8	3.2
Lifetime	55.2	**57.5**	46.2	62.2	69.6	71.4	55.8	48.0	44.0	**53.1**	42.0	62.8	70.0	67.7	56.7	42.6	18.0
Masturbated in front of partner																	
Past month	10.2	**10.7**	10.0	16.6	11.2	13.2	10.3	8.4	5.5	**9.8**	7.2	25.0	19.2	9.2	7.8	1.0	0.7
Past year	20.6	**21.3**	17.0	28.8	25.9	31.0	19.9	12.8	11.7	**19.8**	18.3	40.0	32.4	27.1	16.2	5.7	0.7
Lifetime	41.5	**43.7**	24.8	49.7	56.3	56.2	44.2	42.9	24.1	**39.4**	25.1	53.4	55.3	55.4	42.5	25.3	7.6
Role played with partner																	
Past month	2.2	**2.6**	3.7	7.1	2.3	3.5	2.4	0.6	0.0	**1.7**	3.9	4.2	2.5	1.2	1.1	0.7	0.0
Past year	7.6	**8.1**	12.9	17.1	6.1	10.2	8.5	2.3	1.1	**7.2**	7.6	13.3	13.8	5.3	5.7	4.3	0.9
Lifetime	23.7	**25.8**	19.2	38.7	27.6	35.0	26.6	18.3	12.9	**21.8**	14.7	31.0	33.8	22.3	24.8	13.9	7.1
Playfully whipped or been whipped by partner as part of sex																	
Past month	2.5	**2.2**	4.6	3.8	2.3	3.1	2.1	0.0	0.0	**2.7**	2.3	9.3	5.4	3.3	0.4	0.0	0.0
Past year	6.0	**5.6**	6.9	12.9	5.6	6.3	5.9	3.1	0.0	**6.4**	6.7	15.6	14.1	6.9	3.1	0.6	0.0
Lifetime	15.0	**16.2**	9.2	22.7	17.9	23.8	18.3	13.3	4.1	**13.8**	8.4	30.3	20.4	18.3	10.4	7.2	2.6
Spanked or been spanked as part of sex																	
Past month	8.1	**6.1**	9.2	15.2	12.4	5.7	2.8	0.5	0.3	**10.0**	23.2	25.4	17.9	7.5	4.3	0.7	0.0
Past year	17.2	**14.2**	13.4	39.2	27.5	13.6	8.7	3.8	0.3	**20.0**	40.3	50.8	37.0	16.4	10.2	0.7	0.0
Lifetime	31.9	**29.5**	16.8	49.4	46.0	41.2	25.9	18.3	7.4	**34.1**	46.0	58.8	56.5	39.5	27.3	11.9	5.2

*Chi-squared p-value <0.001

### Sexual behaviors involving enhancement products and/or media

The most prevalent lifetime behaviors in this section were watching sexually explicit videos or DVDs (>70% overall; see Table 3) followed by reading erotic stories (57% for both women and men) and, for men, looking at sexually explicit magazines (79.0%). Significantly more women than men reported having used a vibrator or dildo (50.2% vs. 32.9%). Significantly more men reported having used a phone app related to sex, looked at a sexually explicit magazine, watched a sexually explicit video or DVD, used over the counter enhancement herbs or pills, and having received nude or semi-nude photos of someone.

**Table 3:** Use of enhancement products and media in sexual behaviors by gender and age (N = 2021, Men: n = 975, Women: n = 1046).

			Men (%)								Women (%)						
	Total (%)	Total Men	18–24	25–29	30–39	40–49	50–59	60–69	70+	Total Women	18–24	25–29	30–39	40–49	50–59	60–69	70+
Used a vibrator/dildo*																	
Past month	13.1	**5.5**	3.9	4.2	4.2	9.3	6.8	5.3	2.5	**20.0**	18.5	28.7	29.6	20.4	21.5	12.9	6.5
Past year	22.9	**13.7**	11.3	13.7	15.7	17.9	16.0	10.2	7.5	**31.4**	24.1	41.6	43.1	38.3	35.1	20.4	10.9
Lifetime	41.9	**32.9**	14.9	24.8	40.0	44.0	33.2	37.0	27.5	**50.2**	31.8	56.8	63.8	58.0	58.0	46.2	20.3
Used anal sex toys																	
Past month	3.3	**3.6**	6.4	2.3	5.8	4.1	3.7	0.0	1.7	**3.1**	4.2	5.1	3.7	2.3	2.1	3.5	1.9
Past year	7.5	**7.7**	7.6	11.9	13.8	10.1	6.7	1.4	2.3	**7.3**	8.2	11.0	11.8	9.1	5.1	4.7	1.9
Lifetime	17.2	**18.3**	10.6	18.0	25.9	22.1	19.6	20.0	7.3	**16.1**	8.2	21.5	20.6	21.8	15.0	15.4	5.7
Over the counter sexual enhancement pills or herbal supplement*																	
Past month	2.2	**3.6**	1.4	0.0	3.0	3.5	4.5	5.6	5.9	**0.9**	0.0	3.9	0.0	.5	.9	1.2	0.0
Past year	5.9	**9.2**	5.2	4.7	8.7	8.0	11.7	12.9	11.3	**2.9**	1.3	6.2	3.1	1.6	2.6	4.6	0.0
Lifetime	14.2	**21.2**	7.7	5.9	23.0	18.6	25.0	32.6	29.3	**7.9**	2.6	10.4	8.6	9.3	7.1	11.3	2.7
Read erotic stories																	
Past month	8.5	**8.6**	11.0	4.1	12.3	5.0	11.4	7.4	7.4	**8.4**	17.7	15.1	16.1	3.7	6.2	2.1	3.8
Past year	22.6	**23.0**	28.6	19.2	23.0	23.4	23.3	23.9	18.6	**22.2**	38.4	36.1	32.6	18.0	18.2	9.5	12.7
Lifetime	57.2	**57.2**	44.3	36.6	52.2	60.9	69.6	66.4	56.2	**57.2**	53.6	57.1	59.2	55.4	59.0	58.4	54.7
Read guide book or self-help book about sex																	
Past month	1.0	**1.0**	3.4	3.6	0.0	0.4	0.9	0.0	0.0	**0.9**	0.0	4.9	1.3	0.0	0.0	1.0	0.0
Past year	4.4	**4.6**	11.0	6.2	4.3	4.9	3.8	0.5	2.7	**4.1**	6.5	10.6	5.1	3.5	2.4	3.2	0.0
Lifetime	33.1	**32.0**	20.5	21.1	37.0	36.7	30.5	37.1	36.0	**34.2**	15.4	34.7	38.4	38.7	32.8	38.4	32.4
Used a phone app related to sex*																	
Past month	2.5	**3.3**	3.8	2.2	8.4	4.2	2.1	2.1	0.0	**1.7**	4.8	3.4	4.4	0.5	0.5	0.0	0.0
Past year	4.5	**6.5**	8.4	8.2	10.8	9.4	3.7	4.7	0.6	**2.6**	5.4	6.7	5.7	2.5	0.5	0.0	0.0
Lifetime	8.8	**11.6**	17.4	17.6	17.6	12.8	8.2	7.3	2.0	**6.3**	14.7	12.6	11.0	5.9	3.6	0.5	0.9
Looked through sexually explicit magazine*																	
Past month	4.7	**7.5**	10.2	4.1	5.7	11.5	7.8	8.5	2.1	**2.1**	2.4	6.0	3.7	1.7	0.8	1.2	0.0
Past year	14.0	**22.1**	21.7	29.7	20.1	23.8	19.9	24.7	17.3	**6.4**	6.9	11.2	10.6	8.5	3.8	4.4	0.0
Lifetime	66.2	**79.0**	51.6	71.5	76.6	83.5	88.0	85.3	86.2	**54.4**	23.0	45.2	55.1	58.7	63.2	62.1	54.4
Watched sexually explicit videos or DVDs (porn) *																	
Past month	23.9	**35.3**	39.5	40.5	49.2	37.9	31.0	26.8	22.7	**13.4**	22.1	34.1	21.6	13.8	5.0	5.3	1.0
Past year	38.8	**53.4**	59.6	69.0	59.8	56.1	51.1	45.2	35.9	**25.3**	33.6	51.6	38.3	32.0	15.6	10.9	3.5
Lifetime	70.9	**82.3**	73.3	83.5	84.0	89.7	84.9	83.2	72.0	**60.4**	47.6	61.1	69.6	69.2	66.8	57.7	37.1
Had sex with someone over Facetime/Skype																	
Past month	1.7	**2.4**	4.9	1.1	3.0	3.6	.8	2.4	0.7	**1.0**	5.3	2.6	0.7	0.0	0.0	0.8	0.0
Past year	4.8	**5.5**	10.0	6.8	7.4	6.5	3.6	3.1	1.6	**4.2**	11.3	14.5	4.4	2.2	0.5	1.9	0.9
Lifetime	12.1	**13.9**	15.4	21.2	21.2	19.1	10.5	6.4	3.6	**10.5**	17.0	26.5	16.2	8.3	6.8	3.1	1.8
Sent nude or semi-nude photo of self to someone																	
Past month	5.6	**5.1**	6.0	11.0	6.2	5.6	3.8	1.8	3.2	**6.0**	19.5	10.7	9.2	7.8	1.7	0.0	0.0
Past year	12.9	**12.0**	18.4	21.6	15.8	15.3	8.6	3.4	3.9	**13.7**	29.7	32.6	24.2	14.9	5.7	0.0	0.0
Lifetime	25.5	**24.0**	29.2	38.9	40.3	28.7	17.4	9.8	6.5	**26.9**	49.8	49.3	47.0	28.0	16.6	6.1	5.7
Received nude or semi-nude photo of someone*																	
Past month	6.4	**9.0**	10.8	11.6	11.7	10.3	8.6	4.4	5.2	**4.0**	14.2	8.3	5.7	3.9	1.1	0.0	0.0
Past year	16.3	**21.3**	31.4	39.0	30.7	22.9	15.5	7.4	7.9	**11.7**	33.1	22.3	18.2	12.0	5.6	0.6	1.9
Lifetime	33.7	**41.0**	46.7	62.2	51.4	46.4	36.5	27.3	20.2	**27.0**	48.3	44.4	40.3	33.0	19.4	7.5	7.6
Flirted with someone over SMS/chat																	
Past month	14.2	**14.7**	25.6	28.0	23.4	19.7	5.3	3.4	3.2	**13.8**	35.4	32.6	23.0	11.6	5.6	1.4	0.9
Past year	24.6	**26.7**	47.7	49.3	39.3	34.6	10.8	10.0	4.4	**22.7**	52.2	48.0	34.2	24.6	11.9	4.1	1.8
Lifetime	37.9	**39.9**	55.5	67.2	63.4	53.1	20.8	19.3	8.2	**36.1**	67.7	68.9	55.2	42.4	23.0	10.3	3.9

*Chi-squared p-value <0.001

### Social sexual experiences

Going to a strip club was the most common lifetime “social” sexual experience for both women and men, although significantly more men reported ever having done this (59.4% vs. 30.1%; see Table 4). Similarly, significantly more men reported having ever engaged in a threesome (17.8% vs. 10.3%) or group sex (11.5% vs. 6.3%). Fewer Americans had taken a class or workshop about sex, attended a sex party or swinger’s party, or attended a BDSM party, and no statistically significant differences were found for these latter three items. Fewer than 2% of Americans reported engaging in any individual social sexual behavior in the past month.

**Table 4:** Social sexual experiences by gender and age (N = 2021, Men: n = 975, Women: n = 1046).

			Men (%)								Women (%)						
	Total (%)	Total Men	18–24	25–29	30–39	40–49	50–59	60–69	70+	Total Women	18–24	25–29	30–39	40–49	50–59	60–69	70+
Gone to strip club*																	
Past month	1.1	**1.7**	2.3	3.0	2.3	4.2	0.0	0.0	0.6	**0.4**	0.9	2.3	0.7	0.0	0.0	0.0	0.0
Past year	5.9	**7.9**	11.9	20.1	11.4	10.0	3.3	2.5	0.6	**4.0**	6.7	13.3	9.3	1.2	0.9	0.5	0.0
Lifetime	44.2	**59.4**	20.7	56.6	61.7	73.3	69.0	64.7	55.2	**30.1**	13.7	33.4	39.4	31.3	36.1	31.5	12.5
Taken class/ workshop to learn about sex																	
Past month	0.2	**0.0**	0.0	0.0	0.0	0.0	0.0	0.0	0.0	**0.5**	0.0	4.1	0.0	0.0	0.0	0.0	0.0
Past year	1.2	**1.2**	2.9	5.1	0.8	0.0	0.6	0.0	0.7	**1.4**	0.0	7.9	1.5	1.6	0.0	0.0	0.0
Lifetime	3.6	**3.6**	5.3	5.1	2.9	6.2	2.2	2.2	2.0	**3.5**	1.2	12.0	3.6	4.0	1.9	2.5	0.9
Had a threesome*																	
Past month	0.7	**0.9**	1.5	0.0	0.0	0.6	1.8	0.5	1.4	**0.6**	0.0	3.5	0.7	0.0	0.4	0.0	0.0
Past year	2.3	**3.1**	6.5	4.4	2.9	2.7	2.9	1.6	1.7	**1.5**	0.0	6.1	2.2	0.7	1.0	1.1	0.0
Lifetime	13.9	**17.8**	11.2	16.0	17.3	23.1	21.4	19.2	10.6	**10.3**	2.4	17.7	12.5	14.3	13.7	4.7	3.1
Had group sex*																	
Past month	0.6	**0.7**	1.2	0.0	0.0	0.6	1.2	0.5	1.1	**0.6**	0.0	2.6	0.7	0.0	0.8	0.0	0.0
Past year	2.0	**2.5**	2.6	4.4	2.2	2.7	2.3	2.3	1.7	**1.5**	0.0	4.2	2.3	1.4	1.4	1.1	0.0
Lifetime	8.8	**11.5**	5.1	11.8	11.6	14.9	14.1	13.5	5.7	**6.3**	1.0	11.7	9.2	7.3	9.3	2.2	0.9
Gone to sex party or swinger’s party																	
Past month	0.7	**0.5**	0.0	1.9	0.0	0.7	0.6	0.5	0.3	**0.9**	0.0	7.2	0.7	0.0	0.0	0.0	0.0
Past year	1.9	**2.1**	1.5	5.2	2.4	2.9	1.4	0.5	1.6	**1.7**	0.0	9.2	2.3	0.7	0.5	0.6	0.0
Lifetime	5.8	**6.3**	3.8	6.3	6.0	8.1	7.4	7.5	3.7	**5.2**	0.8	12.0	7.7	3.8	6.7	2.9	1.8
Gone to BDSM party or dungeon																	
Past month	0.3	**0.2**	0.0	0.0	0.0	0.7	0.3	0.0	0.0	**0.5**	0.0	3.5	0.7	0.0	0.0	0.0	0.0
Past year	1.1	**1.0**	1.4	3.2	0.7	1.6	0.7	0.0	0.0	**1.1**	0.0	5.8	2.1	0.7	0.0	0.0	0.0
Lifetime	3.4	**4.3**	3.9	5.6	3.3	6.0	4.8	3.9	1.9	**2.6**	0.0	10.2	2.1	3.6	2.3	0.0	0.9

*Chi-squared p-value <0.001

### Appeal of sexual behaviors

For more than 20 sexual behavior items, there were no statistically significant gender differences in terms of ratings of appeal. These included having sex in a hotel room, giving/receiving massage, role playing, playful biting, spanking, whipping, tying up, reading erotic stories, dirty talk, and blindfolding. The most appealing behaviors were those commonly associated with romance and/or affection (e.g., saying sweet, romantic things during sex, kissing more often during sex, cuddling more often, making the room feel more romantic) (Table 5). Additionally, large proportions of Americans found the following to be somewhat or very appealing: having sex in other parts of the house, having sex more often, and vaginal intercourse.

**Table 5:** Appeal of sexual behaviors by gender.

	Total (%)				Men (%)				Women (%)			
	Very appealing	Somewhat appealing	Not appealing	Not at all appealing	Very appealing	Somewhat appealing	Not appealing	Not at all appealing	Very appealing	Somewhat appealing	Not appealing	Not at all appealing
Worn sexy underwear or lingerie* (Men: n = 943, Women: n = 1019)	27.3	32.5	16.9	23.3	12.2	26.0	26.2	35.5	41.2	38.5	8.2	12.0
Read erotic stories (Men: n = 947, Women: n = 1015)	14.3	33.4	22.8	29.6	10.9	36.1	26.1	27.0	17.4	30.8	19.7	32.1
Watching sexually erotic videos or DVDs (porn)* (Men: n = 945, Women: n = 1013)	18.0	35.5	19.4	27.0	25.1	41.1	16.9	16.8	11.4	30.3	21.8	36.6
Using vibrator/dildo* (Men: n = 946, Women: n = 1013)	17.6	27.3	18.2	36.9	12.2	26.0	21.3	40.5	22.7	28.5	15.3	33.5
Using a penis ring with a partner (Men: n = 943, Women: n = 1020)	6.2	19.4	23.1	51.4	7.2	20.9	22.6	49.3	5.2	17.9	23.5	53.4
Using anal sex toys* (Men: n = 943, Women: n = 1020)	5.0	12.2	16.0	66.9	7.3	14.7	18.5	59.5	2.8	9.9	13.6	73.7
Watching a romantic movie* (Men: n = 947, Women: n = 1018)	31.0	41.6	15.9	11.5	19.2	43.4	23.0	14.3	41.9	40.0	9.3	8.8
Going to a strip club* (Men: n = 945, Women: n = 1020)	6.0	23.0	25.0	46.0	8.0	32.0	26.0	33.9	4.1	14.6	24.2	57.1
Looking at a sexually explicit magazine* (Men: n = 944, Women: n = 1013)	9.6	34.8	26.0	29.7	13.5	44.0	22.8	19.6	5.9	26.1	28.9	39.1
Flirting over text/SMS/SnapChat (Men: n = 940, Women: n = 1013)	14.4	27.3	16.0	42.3	12.9	29.2	18.7	39.2	15.8	25.6	13.6	45.1
Having phone sex with a partner* (Men: n = 944, Women: n = 1024)	7.0	19.8	27.5	45.7	7.3	23.6	28.8	40.3	6.8	16.2	26.3	50.7
Having sex over FaceTime or Skype* (Men: n = 943, Women: n = 1017)	3.7	10.1	20.8	65.4	5.4	11.8	25.4	57.4	2.2	8.5	16.5	72.9
Taking or sharing nude or somewhat nude photos with a partner* (Men: n = 942, Women: n = 1019)	10.6	21.8	21.0	46.6	12.5	26.6	23.1	37.7	8.8	17.4	19.0	54.8
Taking video of yourself masturbating or having sex* (Men: n = 945, Women: n = 1020)	4.7	11.4	18.2	65.7	6.7	13.5	21.4	58.4	2.8	9.4	15.3	72.6
Having sex where someone might see you* (Men: n = 943, Women: n = 1019)	7.2	20.1	23.9	48.9	8.6	23.0	26.2	42.1	5.9	17.3	21.7	55.1
Having sex where someone might hear you* (Men: n = 943, Women: n = 1016)	11.3	25.4	25.4	37.9	12.0	28.8	25.2	34.0	10.7	22.2	25.6	41.4
Tying up your partner or being tied up as part of sex (Men: n = 948, Women: n = 1020)	8.5	20.8	18.9	51.8	8.0	21.5	23.0	47.6	9.0	20.2	15.1	55.7
Masturbating in front of your partner* (Men: n = 947, Women: n = 1018)	11.7	25.9	24.5	37.9	12.9	27.9	24.5	31.8	10.3	23.2	23.9	42.7
Watching your partner masturbate in front of you* (Men: n = 945, Women: n = 1019)	21.1	27.3	19.1	32.5	29.1	31.4	15.2	24.2	13.7	23.5	22.7	40.1
Having your toes/feet sucked/licked by partner or licking/sucking partner’s toes/feet (Men: n = 943, Women: n = 1018)	4.8	10.7	24.8	59.7	5.0	12.1	27.1	55.8	4.7	9.4	22.7	63.3
Playfully whipping or being whipped by a partner as part of sex (Men: n = 941, Women: n = 1020)	5.8	14.5	22.6	57.1	4.5	16.2	25.6	53.7	7.0	13.0	19.8	60.2
Spanking or being spanked as part of sex (Men: n = 938, Women: n = 1019)	8.9	19.5	21.6	50.0	6.7	19.3	27.2	46.8	11.0	19.6	16.4	52.9
Kissing more often during sex (Men: n = 943, Women: n = 1016)	46.1	40.3	7.1	6.6	42.7	43.3	7.5	6.6	49.3	37.5	6.7	6.5
Having rough sex (Men: n = 946, Women: n = 1012)	13.5	27.5	22.6	36.4	14.0	28.1	25.5	32.4	13.0	27.0	19.9	40.2
Saying sweet, romantic things during sex (Men: n = 947, Women: n = 1022)	39.9	41.0	10.2	8.9	32.7	46.7	13.0	7.7	46.6	35.7	7.6	10.1
Having gentle sex (Men: n = 941, Women: n = 1021)	43.9	43.2	5.9	7.0	42.2	45.6	5.4	6.8	45.4	41.1	6.2	7.3
Taking a class or workshop to learn new things about sex (Men: n = 944, Women: n = 1019)	3.0	16.4	28.3	52.3	3.2	16.6	30.5	49.7	2.7	16.2	26.2	54.8
Making the room feel more romantic (Men: n = 943, Women: n = 1021)	33.2	43.9	11.9	10.9	24.4	49.6	14.9	11.1	41.3	38.7	9.2	10.8
Dirty talk (Men: n = 947, Women: n = 1018)	20.4	35.8	20.6	23.2	20.9	39.2	20.1	19.8	20.0	32.6	21.0	26.3
Having a threesome* (Men: n = 948, Women: n = 1019)	7.2	14.9	14.2	63.7	12.3	21.8	16.4	49.4	2.5	8.6	12.1	76.9
Having group sex* (Men: n = 946, Women: n = 1018)	4.7	12.0	14.4	68.9	7.4	18.2	19.2	55.2	2.1	6.3	10.0	81.6
Going to a sex party or swingers’ party* (Men: n = 942, Women: n = 1018)	2.3	9.3	16.3	72.0	3.9	12.6	21.0	62.5	0.9	6.3	12.0	80.9
Going to a BDSM club, party, or dungeon* (Men: n = 941, Women: n = 1020)	1.0	6.0	12.9	80.1	1.1	7.1	15.7	76.0	0.9	5.0	10.2	83.9
Playfully biting, or being bitten, as part of sex (Men: n = 941, Women: n = 1021)	13.7	30.2	20.2	36.0	12.3	32.2	22.6	32.9	14.9	28.3	17.9	38.9
Role playing with a partner (Men: n = 948, Women: n = 1017)	9.5	27.9	26.3	36.3	10.6	29.5	27.2	32.7	8.5	26.5	25.4	39.6
Having anal sex* (Men: n = 942, Women: n = 1018)	9.0	15.4	17.6	57.9	15.3	20.0	18.7	45.9	3.2	11.1	16.7	69.1
Give or receive a massage before sex (Men: n = 942, Women: n = 1021)	40.1	39.2	7.7	12.9	33.8	44.9	8.3	12.9	45.9	34.0	7.2	13.0
Cuddling more often (Men: n = 945, Women: n = 1021)	52.8	35.0	5.6	6.6	41.9	44.3	6.8	7.0	62.8	26.4	4.5	6.3
Having sex more often* (Men: n = 945, Women: n = 1016)	49.0	34.9	8.6	7.6	58.2	32.1	3.7	6.1	40.4	37.5	13.1	9.0
Having sex with a stranger* (Men: n = 937, Women: n = 1023)	6.1	19.0	16.3	58.6	10.3	29.0	18.1	42.5	2.2	9.8	14.7	73.3
Vaginal intercourse (Men: n = 944, Women: n = 1018)	71.3	16.4	3.0	9.2	72.8	13.0	2.6	11.6	69.9	19.7	3.5	7.0
Watching partner undress* (Men: n = 944, Women: n = 1013)	37.0	42.1	10.2	10.7	46.0	40.8	4.6	8.6	28.5	43.3	15.5	12.7
Receiving oral sex* (Men: n = 944, Women: n = 1016)	51.6	27.1	7.6	13.7	60.5	24.2	4.8	10.6	43.3	29.7	10.3	16.7
Giving oral sex* (Men: n = 942, Women: n = 1015)	30.9	35.9	15.5	17.6	41.3	35.2	10.6	12.8	21.3	36.6	20.0	22.1
Having your anus stimulated by your partner’s fingers (Men: n = 944, Women: n = 1020)	9.2	17.9	16.9	56.1	10.4	19.2	17.5	52.9	8.1	16.7	16.2	59.0
Stimulating partner’s anus with your fingers* (Men: n = 945, Women: n = 1020)	9.7	20.3	16.8	53.1	16.2	28.4	16.8	38.6	3.8	12.9	16.8	66.6
Watching others do sexual things* (Men: n = 944, Women: n = 1024)	12.2	27.5	21.2	39.1	16.3	33.4	20.2	30.0	8.5	22.0	22.1	47.5
Having sex in a hotel room (Men: n = 949, Women: n = 1019)	36.2	43.7	10.2	9.8	36.7	46.2	9.0	8.2	35.8	41.4	11.4	11.3
Getting a couples massage* (Men: n = 944, Women: n = 1018)	20.9	35.9	17.2	25.9	15.1	37.5	22.5	24.9	26.3	34.4	12.4	26.9
Being naked in a steam room or jacuzzi with my partner* (Men: n = 945, Women: n = 1019)	31.0	38.2	11.7	19.1	33.9	40.8	10.4	14.9	28.4	35.8	12.9	22.9
Experiencing pain as part of sex* (Men: n = 944, Women: n = 1021)	2.6	8.8	21.0	67.5	1.5	7.0	23.1	68.4	3.6	10.6	19.1	66.8
Blindfolding your partner or being blindfolded by your partner as part of sex (Men: n = 943, Women: n = 1017)	11.5	23.4	21.5	43.5	11.8	24.2	24.1	39.9	11.2	22.8	19.1	46.9
Having sex in other parts of the house (Men: n = 946, Women: n = 1018)	35.3	42.1	10.2	12.3	38.2	41.5	9.3	11.1	32.7	42.7	11.1	13.5

*Chi-squared p-value <0.001

However, there were more than 25 behavioral items that significantly more men rated as appealing compared to women. Among others, these included all anal sexual behaviors (anal sex, anal toys, anal fingering), giving and receiving oral sex, most uses of media related to sex (watching explicit videos/DVDs, phone sex, sex over FaceTime or Skype, sending or receiving nude photos, etc.), having sex where one might be seen or heard by others, watching others (e.g., watching a partner undress, watching a partner masturbate, watching others do sexual things), and group sexual experiences (e.g., threesomes, group sex, sex parties). However, it is important to note that just because men rated these items as more appealing than women does not mean that they were generally appealing. Some behaviors, such as anal intercourse and group sex, were rated as “not appealing” or “not at all appealing” by most men and women.

There were 5 sexual behaviors rated as significantly more appealing by women as compared to men. These were watching a romantic movie, getting a couple’s massage, using a vibrator or dildo, wearing sexy underwear/lingerie, and experiencing pain as part of sex.

### Relationships between appeal and behaviors

After adjusting for age, relationship status, relationship duration, and perceived health status, the appeal of a behavior significantly increased the odds of having recently engaged in the behavior for both men and women for all of the behaviors analyzed (Table 6).

**Table 6:** Relationship between appeal of a behavior and lifetime engagement in the behavior, stratified by gender.

	Men	Women
	*aOR*^1^ *(95% CI)*	*aOR*^1^ *(95% CI)*
Worn sexy underwear or lingerie	8.72 (6.07, 12.52)*	14.31 (8.39, 24.43)*
Read erotic stories	13.50 (8.95, 20.35)*	10.45 (7.02, 15.56)*
Watching sexually explicit videos or DVDs (porn)	15.83 (8.92, 28.10)*	31.58 (18.70, 53.33)*
Using vibrator/dildo	18.42 (12.86, 26.39)*	27.35 (16.21, 46.13)*
Using anal sex toys	11.38 (7.35, 17.62)*	19.57 (9.83, 38.96)*
Going to a strip club	8.20 (5.15, 13.03)*	7.89 (5.51, 11.28)*
Looking at a sexually explicit magazine	6.32 (3.78, 10.56)*	10.76 (6.37, 18.15)*
Flirting over text/SMS/SnapChat	7.59 (4.51, 12.75)*	13.26 (8.34, 21.09)*
Having sex over FaceTime or Skype	11.92 (6.20, 22.91)*	11.40 (5.42, 23.97)*
Tying up your partner or being tied up as part of sex	11.97 (8.21, 17.46)*	11.35 (6.73, 19.16)*
Playfully whipping or being whipped by a partner as part of sex	15.33 (8.75, 26.87)*	12.42 (7.27, 21.21)*
Spanking or being spanked as part of sex	25.49 (15.70, 41.41)*	26.74 (14.41, 49.63)*
Taking a class or workshop to learn new things about sex	4.63 (2.17, 9.87)*	8.60 (3.63, 20.33)*
Having a threesome	8.19 (5.03, 13.33)*	10.94 (5.90, 20.27)*
Having group sex	11.31 (6.19, 20.66)*	12.18 (5.32, 27.90)*
Going to a sex party or swingers’ party	12.43 (6.14, 25.15)*	9.82 (4.13, 23.36)*
Going to a BDSM club, party, or dungeon	30.53 (11.86, 78.54)*	30.01 (7.45, 121.51)*
Role playing with a partner	10.33 (5.84, 18.27)*	9.90 (5.59, 17.53)*
Vaginal intercourse	35.30 (14.65, 85.06)*	66.34 (20.75, 212.12)*
Giving oral sex	33.83 (17.75, 64.47)*	41.99 (14.24, 123.82)*
Receiving oral sex	31.51 (15.36, 64.67)*	23.98 (10.84, 53.04)*
Anal sex	11.85 (7.60, 18.45)*	14.84 (8.57, 25.69)*

^1^Adjusted for age, relationship status, relationship duration, and perceived health status

*p<0.001

## Discussion

This study contributes to our understanding of more diverse adult sexual behaviors than has previously been captured in U.S. nationally representative probability surveys. Sexuality educators and clinicians are often faced with questions from students or patients who want to know whether their sexual interests or behaviors are common or rare and these data will facilitate the answering of such questions (though certainly the prevalence of a behavior is not an indication of whether said behavior is a “good”, “bad”, healthy, or enjoyable behavior for a particular person, dyad, or group). To our knowledge, this paper also includes the first data from a U.S. nationally representative probability sample that describes Americans’ relationships structures (e.g., monogamous, open, etc.).

In comparison to the 2009 NSSHB, the most common lifetime behaviors were the same—solo masturbation for men and vaginal intercourse for women. For masturbation with a partner, we found higher rates for both men and women in the last month and over the lifetime. This may have been influenced by how the questions were asked as the NSSHB asked about “masturbation *with a partner*” and we asked about “masturbation *with someone else*” and “masturbation *in front of* a partner”, which may imply a performer and an observer (emphases added).

The NSHLS found that vaginal sex was one of the behaviors that both men and women found very appealing [35]. In their study, 76% of women and 84% of men reported vaginal sex to be “very appealing” but we found slightly lower rates with 70% of women and 73% of men reporting the same. The reason for this is unclear. It may reflect our study’s inclusion of Americans ages 60s-90s, more of whom may experience vaginal sex as uncomfortable (e.g., due to postmenopausal vaginal dryness) or difficult (e.g., due to erectile dysfunction or, again, vaginal dryness) and thus less appealing. However, our participants rated many more behaviors as appealing compared to vaginal sex. This may be because we included more behaviors that were less genital-focused or less influenced by gender or sexual orientation (e.g. cuddling). Another notable difference is that, in the NHSLS, using a dildo or vibrator was rated as very appealing by only about 4% of participants whereas we found 12.2% of men and 22.7% of women reported the same. This finding is consistent with an increased reporting of sex toy use in the U.S. in at least the past decade [32, 36].

The most appealing behaviors for all participants, regardless of gender, were those commonly associated with romance and affection. Similarly, Joyal, Cossette, and Lapierre [37] included over 1,500 Canadian adults in a non-representative sample study of 55 fantasy themes and found some of the most common fantasies for both men and women included romantic elements. Although fantasies and the appeal of behaviors are similar, they are different in the perceived potential of the behavior—e.g., limiting a behavior to a dream/fantasy versus something one might actually engage in. In addition, other researchers have found a significant and strong relationship between affectionate behaviors and sexual satisfaction.

As some individuals who engage in kink behaviors may experience shame or stigma [38], these data may help to contextualize diverse behaviors as normative in contemporary America, albeit infrequent. As the reported recency of several light kink behaviors (e.g., spanking, tying up, etc.) were not significantly different in their reported prevalence between men and women, this suggests perhaps that at least some of these behaviors are not gendered. We do note, however, the large gaps between many of the sexual behaviors we asked about in terms of their *recency*; that is, about one-third of the sexual behaviors were reported by 1–2% of Americans as having occurred in the previous month even though many more reported having engaged in these same behaviors in their lifetime. Behaviors that more often occurred in Americans’ more distant pasts included sex in public, tying up, foot/toe sucking or licking, role playing, whipping, sex via video, going to a strip club, having a threesome, having group sex, viewing sexually explicit magazines, and reading books about sex. Even anal intercourse was reported by few respondents as having occurred in the past month, though about 10 times as many Americans reported ever having tried it in their lifetime. In contrast, the gap between past month and lifetime behaviors was considerably smaller for vaginal intercourse, oral sex, and masturbation, underscoring these behaviors as some of the most common and characteristic behaviors of Americans’ sex lives. Spanking and vibrator use were also somewhat closer in terms of their recent and lifetime prevalence.

The often sizable differences between recent versus lifetime behaviors indicate some of the more regularly practiced sexual behaviors compared to those that may more often be part of sexual exploration, experimentation, or particular to certain past partners or life stages. Subsequent research might investigate developmental stages in individuals’, couples’ or groups’ sexual lives that may be more or less likely to include varied exploration. For example, it may be that the early months of a couple’s sex life may be marked by experimentation or exploration as sexual partners seek to impress, surprise, seduce, or learn about one another in terms of their emotional and physical responses. It may also be that some individuals engage in more diverse sexual behaviors over time as they become more comfortable exploring together or in response to sexual boredom.

Given that nearly one-third of women and nearly one-quarter of men had not engaged in sexual activity with anyone in the last year (consistent with NSSHB data)–and about 1 in 10 partnered Americans considered themselves monogamous but sexless—it is important to acknowledge that sizable proportions of Americans do not engage in partnered sexual activities during certain periods of their lives. Subsequent research might attend to reasons why people abstain from sex, even when they have romantic relationship partners, as well as the positive and/or negative impacts on their lives and relationships when this occurs.

This study adds to our understanding of diverse human sexual expression. Findings may inform mental and physical health clinicians in ways that improve their practices. Stereotypes that suggest men are uninterested in kissing, cuddling, or other forms of romance may also be challenged by these and other data that demonstrate the value of romantic and affection behaviors to both women and men [39]. Clinicians in various settings working with adults in the U.S. can better meet the needs of their clients by being more aware of the diversity of sexual practices across the lifespan.

### Strengths and limitations

Because we aimed to make population estimates, we utilized U.S. nationally representative probability sampling and worked with one of the few companies equipped to carry out such surveys in the United States. The present survey occurred via the Internet, which may have enhanced the valid reporting of sensitive and/or stigmatizing sexual behaviors. Even so, American culture continues to be marked by stigma, shame, and taboo around certain aspects of sexuality and thus a limitation is that some people may have felt this stigma and thus chosen to not participate in our study or to not provide accurate reports. Then again, it is possible that some people—in response to stigma or shape—felt encouraged by a chance to report openly about their sexual lives in a confidential survey.

Additional strengths are that KnowledgePanel^®^ members are practiced at taking Internet-based surveys and thus familiar with how to respond to online questionnaires and that panel members also tend to complete most items with low per-item refusal rates. The reported prevalence of oral sex, vaginal intercourse, and anal intercourse was remarkably consistent with other recent U.S. nationally representative probability surveys (i.e., the various waves of the NSSHB), which suggest that respondents to the present survey were not likely to have been particularly different from those participating in other similar surveys of sexual behavior. Similarly, the distribution of sexual orientation categories is similar to that of the NSSHB.

As happens with most studies, we were limited by time and funding and thus had to make difficult choices about which items to include. We asked about a far greater of sexual behaviors than other national probability surveys (whether in the U.S., U.K., Australia, or Finland), but still could not ask about every sexual behavior of interest to our research team or stakeholders. We also did not differentiate between whether people had been the giver or receiver of certain behaviors (for example, whether they had spanked or been spanked; these were combined in a single item) and we left some descriptions open to subjective interpretation. For example, people may have varying ideas about what constitutes “rough sex” or “gentle sex” or saying “dirty things” or “sweet, romantic things” during sex. Although this is a limitation of our current research, it is similarly a limitation of most research that assesses seemingly banal behaviors such as vaginal intercourse or masturbation, given the many different ways each can be enacted and interpreted. Further, because we did not require information about the specific kinds of phone apps related to sex that respondents may have used, we simply inquired about their use of “phone apps related to sex”. Had we asked specifically about the kinds of phone apps related to sex (e.g., Grindr, Tinder, or other apps for dating or meeting for sexual encounters; sex education apps; or apps featuring erotica) we would have had a more detailed understanding about respondents’ use of technology related to sexualities.

The inability to clarify meaning to respondents or to ask them for their interpretation of items is a limitation of survey research. However, our data provide insights for subsequent research to approach with greater detail. Similarly—and consistent with the Australian Survey of Health and Relationships [16]—we were not able to take a detailed look at, for example, the gender make-ups of respondents’ threesomes or group sex experiences, or what proportion of respondents’ experiences of specific behaviors were wanted, desired, or consensual. Our research is also limited to American women and men and to adults; thus, we make no claims about sexual behaviors among individuals in other cultures or even among younger U.S. adolescents. As with most U.S. probability surveys, our sample was also limited to non-institutionalized individuals and to those who can read and respond to questions written in English.

We feel that this study makes an important contribution to the literature by extending the sexual behaviors most commonly assessed. Although we asked about sexual behaviors associated with risk of pregnancy and infection we mostly asked about sexual behaviors that may be considered as being about exploration, recreation, affection, or novelty (e.g., spanking, whipping, kissing, saying romantic or “dirty” things during sex). Given the large amount of data presented in the current paper, analyses are presented only by gender and age and not, for example, by self-identified sexual orientation or other background characteristics such as education, race/ethnicity, relationship status, or sexual experience. Future manuscripts will be able to address the existing data in a more detailed manner.

## Conclusions

Overall, findings add to our understanding of more diverse U.S. adult sexual behaviors and the appeal of a range of sexual behaviors. Findings provide baseline rates for a wide array of sexual behaviors among adults in the general populations in the United States for which such estimates have been previously absent. For practitioners and providers, this information may assist in meeting the needs of diverse populations including improved information exchange and educational efforts.
